# Fruits and vegetables intake improves birth outcomes of women with gestational diabetes mellitus and hypertensive disorders of pregnancy

**DOI:** 10.1186/s40795-023-00814-w

**Published:** 2024-01-02

**Authors:** Christian Sewor, Akua A. Obeng, Sebastian Eliason, Evans K. Agbeno, A. Kofi Amegah

**Affiliations:** 1 Public Health Research Group, Department of Biomedical Sciences, University of Cape Coast, Private Mail Bag, Cape Coast, Ghana; 2 Department of Public Health and Community Medicine, Central University of Kerala, Kasaragod, Kerala India; 3 Department of Community Medicine, School of Medical Sciences, University of Cape Coast, Cape Coast, Ghana; 4 Department of Obstetrics and Gynecology, School of Medical Sciences, University of Cape Coast, Cape Coast, Ghana

**Keywords:** Hypertensive disorders of pregnancy, Gestational diabetes mellitus, Low birthweight, Preterm birth, Fruits, Vegetables

## Abstract

**Background:**

Disorders of pregnancy such as hypertensive disorders of pregnancy (HDP) and gestational diabetes mellitus (GDM) have been associated with adverse birth outcomes. However, the ameliorating role of maternal nutrition in the relationship between disorders of pregnancy and adverse birth outcomes has received very little attention. We investigated the relationship between HDP and GDM, and adverse birth outcomes in a Ghanaian population and evaluated the effect modifying role of fruits and vegetables consumption in the relationship.

**Methods:**

We conducted a cross-sectional study among 799 mothers who had recently delivered singletons in the Cape Coast Metropolis, Ghana. Information on HDP, GDM and birth outcomes were retrieved from the maternal health book of the mothers. A food frequency questionnaire was used to assess fruits and vegetables intake during pregnancy. Modified Poisson regression was used to investigate the association between pregnancy disorders, and preterm birth (PTB) and low birth weight (LBW). Stratified analysis was used to assess the effect modifying role of fruits and vegetables consumption in the relationship.

**Results:**

The proportion of mothers with HDP and GDM was 11.3% and 7.5%, respectively. The proportion of the mothers with both conditions was 0.9%. The prevalence of PTB and LBW in the population was 27.9 and 7.3%, respectively. These disorders of pregnancy were associated with increased risk of PTB (Adjusted Prevalence Ration [APR] = 3.02; 95% CI: 2.42, 3.77) and LBW (APR = 5.32; 95% CI: 3.19, 8.88). In the stratified analysis, risk of PTB was higher among mothers classified in tertile I compared to mothers classified in tertiles II and III. For LBW, the risk increased with increasing fruits and vegetables consumption. The interaction p values were 0.0043 and 0.1604 for PTB and LBW, respectively.

**Conclusions:**

We found mothers who were diagnosed with GDM and HDP to have increased risk of delivering a PTB and LBW baby. We also found fruits and vegetables consumption to modify the observed relationship. Mothers diagnosed with GDM and HDP should be advised during antenatal care visits to increase intake of fruits and vegetable consumption to help safeguard their health and that of the developing foetus.

**Supplementary Information:**

The online version contains supplementary material available at 10.1186/s40795-023-00814-w.

## Background

Pregnancy disorders such as hypertensive disorders of pregnancy (HDP) and gestational diabetes mellitus (GDM) are the leading causes of maternal and perinatal mortality [1, 2]. The incidence of HDP has increased significantly over the years in low- and middle-income countries (LMICs) with the highest levels reported in Western and Eastern Africa, and South-East Asia [3]. In 2019, 28,000 deaths were associated with HDP with the highest burden being reported in Western, Eastern and Central Africa [3]. A similar situation has been reported for GDM with a global prevalence of 14% and with the highest prevalence noted in LMICs [4]. The prevalence of GDM in Africa has been estimated to be 14.7% and is higher than the global average [4]. In Ghana, there are no nationally representative estimates for these conditions. However, cross-sectional studies conducted in some geographical areas of the country have reported the prevalence of HDP and GDM to be 21.4% [5] and 9.3% [6] respectively. Pregnancy disorders do not only pose deleterious effects on mothers but also on the developing fetus. Several systematic reviews and meta-analyses have associated maternal disorders of pregnancy with a number of adverse perinatal outcomes including low birth weight (LBW) and preterm birth (PTB) [7–10]. For instance, Mersha et al. [7] found LBW to be prevalent in more than a quarter of mothers who were diagnosed with HDP (37%) whereas Ye et al. [10] found GDM to be associated with increased odds of PTB (OR = 1.51; 95% CI: 1.26, 1.80) and LBW (OR = 1.40; 95% CI: 0.12 to 16.73) among mothers who did not use insulin.

Globally, PTB and LBW are the first and second most common causes of neonatal morbidity and mortality, respectively [11]. In fact, the prevalence of PTB and LBW have been observed to be highest in sub-Saharan African countries and other developing countries [12]. In Ghana, the 2014 Demographic and Health Survey [13] and a global study [12] found the prevalence of LBW and PTB in the country to be 10% and 14.5%, respectively. These adverse perinatal outcomes threatens the survival of newborns owing to the associated increased risk of infections, delayed growth and development, and metabolic and chronic disease predisposition [14].

Pregnancy disorders have been linked to adverse birth outcomes due to the similarities in their biological pathway with both conditions associated with pathophysiological processes such as oxidative stress and inflammation [9, 15]. The similarities in their biological mechanisms has meant that interventions that affect these biological pathways could affect the relationship between pregnancy disorders and birth outcomes. Maternal dietary pattern has been identified as a key factor that affects both maternal and neonatal health. Even though the evidence on how maternal dietary pattern affects adverse maternal and birth outcomes are limited, consumption of fruits and vegetables has been identified to affect oxidative stress and inflammation pathways which tend to play a significant role in the maternal disorders of pregnancy – adverse birth outcome relationship [16]. It has been observed that mothers who eat plenty fruits and vegetables have a reduced risk of experiencing pregnancy disorders and giving birth to preterm babies [16].

We investigated the relationship between HDP/GDM and adverse birth outcomes in a Ghanaian population and evaluated the potential role of fruits and vegetables consumption in ameliorating the relationship. Evidence from this study will help to better tailor nutrition interventions among pregnant women and address the rising incidence of adverse maternal and birth outcomes in Ghana and other developing countries.

## Methods

Data from the Household Air Pollution Exposure and Maternal Disorders of Pregnancy (HAEM) study, was analysed for this study. The study protocol has been described elsewhere [17]. In brief, HAEM was a cross-sectional study conducted among mothers who had recently given birth and attending postnatal clinics of the two main health facilities in the Cape Coast metropolis - Teaching Hospital and University Hospital. The study was conducted from June to August 2020. Nine hundred mothers who had recently given singleton births were randomly sampled to participate in the study with 799 mothers agreeing to participate in the study (response rate 88.8%). We excluded mothers who had been diagnosed of diabetes mellitus or hypertension prior to the index pregnancy from the study. This information was recorded in the mothers’ maternal health book.

### Determinants and outcomes of interest

The determinants of interest were the pregnancy disorders i.e. gestational diabetes mellitus, eclampsia, preeclampsia and gestational hypertension. These conditions were physician-diagnosed and were recorded in the mothers’ maternal health books. Diagnosis of both conditions were made throughout the period of pregnancy with mothers having to present with symptoms or identified as having a high risk in the case of GDM. The outcomes of interest were LBW and PTB. LBW was defined as birth weight less than 2500 g at birth [18] whereas PTB was defined as a live birth before 37 completed weeks of gestation [19]. The birth outcomes were also recorded in the mothers’ maternal health books. We collected information on these determinants of interest during the interviews with the mothers.

### Assessment of fruits and vegetables consumption

A food frequency questionnaire (FFQ) was used to establish the frequency of consumption of fruits and vegetables during the period of pregnancy. In the FFQ, frequency of consumption of fruits and vegetables was assessed generally with no listing of specific fruits and vegetables, and separately for the two food groups on a 9-level scale from “never” to “more than 3 times per day”. This information was used to estimate the fruits and vegetables consumption scores of the mothers.

The consumption scores were estimated as follows: Step 1 involved assigning a score to the frequency of consumption of fruits and vegetables. The score ranged from 0 to 4 for each and was assigned to never (0), once per month (0.036), 2–3 times per month (0.089), once per week (0.143), 2–3 times per week (0.358), 4–5 times per week (0.644), once per day (1.0), 2–3 times per day (2.5), and more than 3 times per day (4.0), respectively. Step 2 involved adding up the frequency scores assigned in step 1 to obtain the total consumption scores which ranged from 0 to8. The consumption scores were then stratified by tertiles.

### Statistical analysis

The demographic and background characteristics, maternal health characteristics, and fruits and vegetables consumption levels of the study participants was presented as frequencies and proportions. Modified Poisson regression with logarithmic link function was used to estimate the effects of HDP/GDM on the occurrence of PTB and LBW. The 95% confidence interval (CI) of the prevalence ratios (PR) estimated from the Modified Poisson regression were based on robust error variance [20]. The following potential confounders were controlled for in the analysis; area of residence, age, educational level, marital status, ethnicity, religion and occupation of mother, gravidity, trimester of first antenatal visit, number of antenatal care visits, type of nutritional supplement taken during pregnancy, and pre-pregnancy BMI.

We stratified the analysis according to tertiles of fruits and vegetables intake to explore the effect modifying role of fruits and vegetables consumption on the observed association. We tested for interaction between diagnosis of pregnancy disorders and intake of fruits and vegetables by entering interaction terms into the models and performing the likelihood-ratio test. Stata version 16 was used to perform the analysis.

## Results

Tables 1 and 2, and their summaries have been published [17]. In brief, about 76% of the participants reside in low-income neighborhoods with 12.0% residing in high-income areas. About 69% of the participants were within the age group of 20–29 years. About 31% of the participants were educated up to university/tertiary level. Participants with no formal education constituted 1.3%. About 89% and 82% of the study participants were Christians and Akans, respectively. Office/administrative workers constituted 23% of the study participants with housewives/unemployed making up 5%.

**Table 1:** Demographic and background characteristics of study participants (n = 799)

Characteristics	Total (n = 799) n (%)	Mothers with HDP/GDM (n = 157) n (%)	Mothers with no HDP/GDM (n = 642) n (%)
**Area of residence**			
Low-income	604 (75.6)	123 (78.3)	481 (74.9)
Middle-income	99 (12.4)	21 (13.4)	78 (12.2)
High-income	96 (12.0)	13 (8.3)	83 (12.9)
**Age group (years)**			
< 20	17 (2.1)	6 (3.8)	11 (1.7)
20–29	550 (68.8)	96 (61.2)	454 (70.7)
30–39	220 (27.5)	50 (31.9)	170 (26.5)
> 39	12 (1.5)	5 (3.2)	7 (1.1)
**Education level**			
No formal	10 (1.3)	3 (1.9)	7 (1.1)
Primary	36 (4.5)	6 (3.8)	30 (4.7)
Junior high	176 (22.0)	45 (28.7)	131 (20.4)
Senior high/secondary/technical	333 (41.7)	62 (39.5)	271 (42.2)
University/tertiary	244 (30.5)	41 (26.1)	203 (31.6)
**Religion**			
Christian	714 (89.4)	132 (84.1)	582 (90.7)
Moslem	83 (10.4)	23 (14.7)	60 (9.4)
Other	2 (0.3)	2 (1.3)	0 (0.0)
**Ethnicity**			
Akan	655 (82.0)	116 (73.9)	539 (84.0)
Ewe	47 (5.9)	14 (8.9)	33 (5.1)
Ga	18 (2.3)	5 (3.2)	13 (2.0)
Hausa	9 (1.1)	0 (0.0)	9 (1.4)
Other northern tribe	70 (8.8)	22 (14.1)	48 (7.5)
**Marital status**			
Single	28 (3.5)	7 (4.5)	21 (3.3)
Married	672 (84.1)	135 (86.0)	537 (83.6)
Cohabiting	98 (12.3)	15 (9.6)	83 (12.9)
Divorced	1 (0.1)	0 (0.0)	1 (0.2)
**Occupation**			
Hairdresser	150 (18.8)	36 (22.9)	114 (17.8)
Seamstress	175 (21.9)	18 (11.5)	157 (24.5)
Fish monger/farmer	19 (2.4)	7 (4.5)	12 (1.9)
Street vendor/hawker	34 (4.3)	5 (3.2)	29 (4.5)
Shop owner/attendant	163 (20.4)	27 (17.2)	136 (21.2)
Office/administrative worker	183 (22.9)	41 (26.1)	142 (22.1)
Nurse/midwife	20 (2.5)	6 (3.8)	14 (2.2)
Teacher	7 (0.9)	0 (0.0)	7 (1.1)
Housewife/unemployed	40 (5.0)	16 (10.2)	24 (3.7)
Other	8 (1.0)	1 (0.6)	7 (1.1)

**Table 2:** Maternal health characteristics of study participants and outcomes of birth (n = 799)

Characteristics	Total (n = 799) n (%)	Mothers with HDP/GDM (n = 157) n (%)	Mothers with no HDP/GDM (n = 642) n (%)
**Pre-pregnancy BMI**			
Underweight (< 18.5 kg/m^2^)	4 (0.5)	0 (0.0)	4 (0.6)
Normal (18.5–24.9 kg/m^2^)	101 (12.6)	13 (8.3)	88 (13.7)
Overweight (25.0–29.9 kg/m^2^)	307 (38.4)	65 (41.4)	242 (37.7)
Obesity (> 29.9 kg/m^2^)	378 (47.3)	78 (49.6)	300 (46.7)
Missing	9 (1.1)	1 (0.6)	8 (1.2)
**Gravida status**			
Gravida 0	418 (52.3)	74 (47.1)	344 (53.6)
Gravida 1	176 (22.0)	42 (26.8)	134 (20.9)
Gravida 2	173 (21.7)	38 (24.2)	135 (21.0)
Gravida 3+	32 (4.0)	3 (1.9)	29 (4.5)
**Trimester of first antenatal care visit**			
First	552 (69.1)	132 (84.1)	420 (65.4)
Second	243 (30.4)	25 (15.9)	218 (34.0)
Third	4 (0.5)	0 (0.0)	4 (0.6)
**No. of antenatal care visit**			
< 8 visits	668 (83.6)	129 (82.2)	539 (84.0)
> 8 visits	131 (16.4)	28 (17.8)	103 (16.4)
**Intake of nutritional supplement**			
Yes	787 (98.5)	154 (98.1)	633 (98.6)
No	12 (1.5)	3 (1.9)	9 (1.4)
**Type of nutritional supplement**			
Iron only	38 (4.8)	4 (2.6)	34 (5.4)
Folate only	176 (22.4)	51 (33.1)	125 (19.8)
Iron and Folate only	489 (62.1)	80 (52.0)	409 (64.6)
Multi – vitamins & minerals	84 (10.7)	19 (12.3)	65 (10.3)
**Regularly taking nutritional supplements**			
Yes	776 (98.6)	153 (99.4)	623 (98.4)
No	11 (1.4)	1 (0.7)	10 (1.6)
**Type of pregnancy disorder**			
Gestational diabetes alone	60 (7.5)		
Hypertensive disorders alone	90 (11.3)		
Both disorders	7 (0.9)		
**Birth outcomes**			
Low birth weight	58 (7.3%)	29 (18.5)	29 (4.5)
Preterm birth	223 (27.9)	93 (59.2)	130 (20.3)

About 86% of the mothers were either overweight or obese. Mothers with no previous pregnancy history constituted 52.3%. The proportion of mothers with three or more previous pregnancies was 4.0%. The proportion of mothers who reported visiting ANC less than eight times during the pregnancy was 84%. Almost (99%) all the mothers reported taking nutritional supplements during pregnancy and regularly. The proportion of mothers with hypertensive disorders of pregnancy and gestational diabetes was 11.3% and 7.5%, respectively with 0.9% of the mothers having both conditions. The prevalence of PTB and LBW in the population was 27.9 and 7.3%, respectively.

The proportion of study participants consuming fruits and vegetables at least once per day was 37.9% and 58.3%, respectively. The proportion of study participants consuming both fruits and vegetables at least once per day was 35.4%. Table 3 presents tertiles of fruits and vegetables consumption of the mothers and according to the outcomes of birth. About 44% of the mothers were classified within the third tertile of fruits and vegetables consumption. The proportion of mothers who were diagnosed with HDP/GDM during pregnancy and those who were not, and classified in the third tertile of fruits and vegetables intake was about the same (43% and 45%, respectively). The highest proportion (43%) of LBW babies were delivered to mothers classified in the first tertiles of fruits and vegetables intake. Close to half (49%) of PTB babies were delivered to mothers classified in the third tertile of fruits and vegetables intake.

**Table 3:** Tertiles of fruits and vegetables consumption of study participants and according to the outcomes of birth (n = 799)

Fruits and vegetables consumption scores (mean ± SD)	Total no. of mothers (n = 799) n (%)	Mothers with HDP/GDM (n = 157) n (%)	Mothers with no HDP/GDM (n = 642) n (%)	LBW (n = 58) n (%)	PTB (n = 223) n (%)
I (0.77 ± 0.31)	210 (26.3)	51 (32.5)	159 (24.8)	25 (43.1)	51 (22.9)
II (1.83 ± 0.64)	225 (28.2)	38 (24.2)	187 (29.1)	12 (20.69)	60 (26.9)
III (3.65 ± 0.78)	354 (44.3)	67 (42.7)	287 (44.7)	21 (36.2)	109 (48.9)
Missing	10 (1.3)	1 (0.6)	9 (1.4)		3 (1.3)
**Fruits consumption scores (mean ± SD)**					
I (0.31 ± 0.11)	216 (27.0)	53 (33.8)	163 (25.4)	18 (31.0)	50 (22.4)
II (0.64 ± 0.00)	271 (33.9)	39 (24.8)	232 (36.1)	12 (20.7)	63 (28.3)
III (1.29 ± 0.67)	303 (37.9)	64 (40.8)	239 (37.2)	28 (48.3)	107 (48.0)
Missing	9 (1.1)	1 (0.6)	8 (1.2)		3 (1.3)
**Vegetables consumption scores (mean ± SD)**					
I (0.27 ± 0.13)	154 (19.3)	30 (19.1)	124 (19.3)	23 (39.7)	35 (15.7)
II (0.74 ± 0.16)	236 (29.5)	50 (31.8)	186 (29.0)	12 (20.7)	69 (30.9)
III (2.54 ± 0.25)	327 (40.9)	77 (49.0)	327 (50.9)	23 (39.7)	119 (53.4)
Missing	5 (0.6)		5 (0.8)		

*SD* standard deviation

Table 4 presents prevalence ratios and their corresponding 95% confidence intervals from Poisson regression with robust error variance regressing PTB and LBW on maternal disorders of pregnancy. We further stratified the estimates according to tertiles of fruits and vegetables intake. Mothers who were diagnosed with GDM/HDP had 3.02 (Adjusted Prevalence Ratio [APR] = 3.02; 95% CI: 2.42, 3.77) increased risk of delivering a PTB baby and 5.32 (APR = 5.32; 95% CI: 3.19, 8.88) increased risk of delivering a LBW baby compared to mothers who were not diagnosed of GDM/HDP. In the stratified analysis, the risk of PTB was higher among mothers classified in tertile I compared to mothers classified in tertiles II and III but with the risk ratio slightly higher among mothers classified in tertile III compared to tertile II (PTB – T1: PR = 7.50, 95% CI: 4.69, 11.98; T2: PR = 2.24, 95% CI: 1.36, 3.68; T3: PR = 2.56, 95% CI: 1.86, 3.51. For LBW, the risk increased with increasing tertiles of fruits and vegetables intake (T1: PR = 5.07, 95% CI: 2.23, 11.54; T2: PR = 5.94, 95% CI: 1.05, 33.71; T3: PR = 7.39, 95% CI: 2.60, 20.95). The interaction p values were 0.0043 and 0.1604, for PTB and LBW, respectively.

**Table 4:** Association of maternal disorders of pregnancy (HDP/GDM) with adverse birth outcomes stratified according to tertiles of fruits and vegetables intake (n = 777)

Characteristics	All sample APR (95% CI)	Fruits and vegetables intake			Interaction *p*-value
		Tertile I APR (95% CI)	Tertile II APR (95% CI)	Tertile III APR (95% CI)	
Preterm birth	3.02 (2.42, 3.77)	7.50 (4.69, 11.98)	2.24 (1.36, 3.68)	2.56 (1.86, 3.51)	0.0043
Low birth weight	5.32 (3.19, 8.88)	5.07 (2.23, 11.54)	5.94 (1.05, 33.71)	7.39 (2.60, 20.95)	0.1604

Model adjusted for area of residence; age, educational level, marital status, ethnicity, religion and occupation of mother; gravidity; trimester of first antenatal visit, number of antenatal care visits, type of nutritional supplement taken during pregnancy, and pre-pregnancy BMI

Tertile values reported are means and standard deviation

*APR* adjusted prevalence ratio, *CI* confidence interval

## Discussion

HDP and GDM were found to be associated with increased risk of PTB and LBW in the study area. Increasing fruits and vegetables consumption during pregnancy was also found to ameliorate the HDP/GDM – PTB relationship but worsen the HDP/GDM – LBW relationship.

### Synthesis with previous evidence

We found mothers who were diagnosed of either GDM or HDP during pregnancy to have increased risk of delivering PTB or LBW baby. Our findings are consistent with the findings of studies from several geographical regions on the topic. Several systematic reviews and meta-analyses have attempted to summarize the growing evidence on the topic and have associated HDP [7, 8, 21–24] and GDM [25–30] with adverse birth outcomes. Bramham et al. [21] reported an incidence rate of 28.1% and 16.9% for PTB and LBW, respectively, among women who were diagnosed with chronic hypertension during pregnancy. Al Khalaf et al. [23] found maternal chronic hypertension to be associated with increased odds of PTB and LBW delivery. A systematic review of cohort studies by Li et al. [24] also found mothers with HDP to have increased odds of having an unfavourable birth outcome including LBW and PTB. Gemechu et al. [22] reviewed studies conducted in Sub-Saharan Africa and also found women with HDP to have increased odds of delivering a LBW (OR = 3.2; 95%CI: 2, 5) and PTB (OR = 7.8; 95% CI; 2.5, 25.3) baby. A review of studies conducted in Ethiopia reported a high prevalence of LBW (37%) among women who were diagnosed with HDP [5]. Our findings on HDP are consistent with the findings of these studies.

Our findings on GDM were also consistent with the findings of previous studies. Wang et al. [30] for instance, reported the pooled median incidence of PTB and LBW among mothers with GDM in low- and middle-income countries to be 6.9% (IQR; 3.3–9.8) and 6.9% (IQR; 3.2–8.3) respectively. A review of studies conducted in Ethiopia [25] also found women who diagnosed with GDM to have significantly higher odds (OR = 10.51; 95% CI = 5.90, 15.12) of having unfavourable birth outcomes including LBW and PTB. A recent systematic review by Bidhendi Yarandi et al. [26] also found women who diagnosed with mild GDM to be at increased risk of delivering a PTB baby (pooled RR = 1.4, 95% CI: 1.1–1.7). A review that focused on studies conducted in South Asia by Mistry et al. [27] also found GDM to be associated with adverse birth and neonatal outcomes including LBW, macrosomia, neonatal hyperglycaemia and intrauterine growth retardation.

In this study, about 86% of the mothers were either overweight or obese, a condition which has been reported as prevalent among pregnant women in Ghana [31, 32] with the risk factors identified as age, socioeconomic deprivation, poor dietary pattern (consumption of high caloric foods) and physical inactivity [33–35].

In this study, we found the prevalence of fruits and vegetables consumption among the mothers to be relatively low. Studies conducted in Ghana have also reported similar findings [36, 37]. Azupogo et al. [36] reported that, vegetable intake among women in Northern Ghana was lower than WHO [38] recommended daily intake of at least 400 g per day. Similarly, a study conducted by Moss and Mushtaq [37] among 74 Ghanaian women reported 69% of the study participants to have low fruit and vegetable consumption patterns. In Ghana, low consumption of fruits and vegetables has been reported to be driven by a myriad of contextual and socioeconomic factors including age and household wealth [39, 40]. The poor food environment in many neighbourhoods characterized as low access to healthy, affordable foods owing to high prices of fruits and vegetables, influx and aggressive marketing of sugar-sweetened beverages, and influx of western fast food chains is another very important reason.

A number of studies have shown that fruits and vegetables consumption during pregnancy can play a critical role in reducing the risk of PTB, LBW, and disorders of pregnancy [16, 41–45]. Kibret et al. [16] found maternal dietary patterns including frequent intake of vegetables, fruits, legumes and whole grains to be associated with decreased odds of pre-eclampsia, GDM and PTB. Another systematic review by Gete et al. [43] also reported dietary pattern during pregnancy which was characterized by high consumption of vegetables, fruits, whole grains, fish and dairy products to be associated with a lower risk of PTB. Maternal intake of fruits and vegetables has also been associated with reduced risk of pre-eclampsia in nulliparous women [41] and PTB [44], and improved birth anthropometrics such as birth weight and birth length [45]. Englund-Ogge et al. [42] also found a dietary pattern that included consumption of vegetables, fruits, oils, whole grain cereals, and fibre-rich bread to be associated with reduced risk of PTB.

A number of studies have found fruits and vegetables consumption during pregnancy to be independently associated with reduced risk of pregnancy disorders and adverse birth outcomes [16, 41–45]. Our study, however, provides important evidence on the effect modifying role of fruits and vegetables consumption in the disorders of pregnancy and adverse birth outcomes relationship. High fruits and vegetables intake, however, appears to increase the risk of LBW and is quite surprising. Misclassification from the crude measurement of fruits and vegetables intake could explain our findings. We collected information on the frequency of consumption of fruits and vegetables during the period of pregnancy in a food frequency questionnaire and summarized the information to estimate consumption levels of the study participants. However, to accurately estimate food intake of individuals, information on the amount of food consumed is also required and should to be addressed in future studies.

### Biological plausibility

The mechanism by which fruits and vegetables consumption could ameliorate the impact of maternal disorders of pregnancy on birth outcomes is likely to be through their effect on oxidative stress. It has been shown that, the antioxidants found in fruits and vegetables can help reduce oxidative stress during pregnancy [46]. For instance, increasing fruits and vegetables intake has been reported to reduce the risk of pre-eclampsia [41, 47] with the risk reduction occurring through mitigation of oxidative stress which underlies preeclampsia [48]. In a prospective cohort study conducted in Korea, maternal vitamin C levels were inversely associated with urinary levels of malondialdehyde, a key biomarker used to assess maternal oxidative stress [45].

Also, micronutrients such as vitamins, polyphenols (flavonoids), essential minerals (potassium, magnesium) and fibre which are readily available in fruits and vegetables are known to facilitate glycaemic and blood pressure control [49–53]. Polyphenols such as flavonoids and antioxidant such as carotenoids, vitamin C and E, have been documented to improve insulin sensitivity by reducing oxidative stress which has been found to inhibit cellular glucose uptake [49]. Dietary fibre has been found to improve insulin sensitivity by delaying the absorption of carbohydrates and increasing insulin secretion [49, 50]. Through these biological pathways, increased fruits and vegetables intake, could potentially help to reduce the adverse effect of GDM on foetal health and development.

Nutrients such as potassium, magnesium, vitamin C, folic acid, flavonoid, and carotenoid which are readily available in fruits and vegetables have also been reported to facilitate the reduction of blood pressure by reducing oxidative stress, improving endothelial function and helping induce vasodilation [51–53]. Macready et al. [54] reported that, polyphenols such as flavonoids can increase endothelium-dependent microvascular reactivity and plasma nitric oxide (NO), as well as reducing C-reactive protein and E-selectin and helping to induce vaso-relaxation and reducing blood pressure as a result. 

Additionally, the antioxidant activity of fruits and vegetables renders them as potent anti-inflammatory foods which helps to decrease inflammation, a key hallmark of the spectrum of adverse pregnancy and birth outcomes [55–58].

### Validity issues

Issues relating to sampling and sample size, generalizability of the study findings has been reported elsewhere [17]. In summary, the random sampling approach and high response rate achieved in the study (89%) minimizes selection bias. The GDM and HDP were diagnosed by qualified physicians with the information gathered from the maternal health book of the mothers. Information on the study outcomes were also recorded from the maternal health books. The potential for exposure and outcome misclassification were therefore minimised in the study.

A food frequency questionnaire (FFQ) was used to establish the frequency of consumption of fruits and vegetables during pregnancy. Using FFQ for estimating usual food and nutrient intake of study participants has well documented limitations. Recall bias is a particular concern and can result in either under- or over-estimation of fruits and vegetables intake. In spite of the limitations, FFQ is the standard tool for measuring diet in epidemiological studies. This is because, according to Kristal et al. [59] FFQ is the only dietary measure that minimizes the very high intra-individual, day-to-day variability in nutrient intake without relying on a multiple day assessment of actual foods consumed. The author further points out that, FFQ is the only feasible method in traditional case-control studies and cross-sectional studies where the usual food intake is ascertained retrospectively.

Use of cross-sectional study design makes it difficult to establish temporal sequence. However, in this study it was obvious from the maternal health records that, diagnosis of disorders of pregnancy among the mothers preceded the birth outcomes. Temporality should therefore not be a problem in this study. We controlled for the confounding effect of several socio-demographic and maternal healthcare factors as well as pre-pregnancy BMI. These covariates including maternal overweight/obesity were associated with both HDP/GDM and the study outcomes.

The likelihood-ratio test was used to conduct a test for interaction in order to evaluate statistically significant subgroup differences. For LBW, the p value was greater than the α level, set at 0.05, and as a result, fails to confirm the observed subgroup differences. However, for PTB, the p value was less than the α level and confirms the observed subgroup differences.

## Conclusions

We found GDM and HDP to be associated with PTB and LBW in this Ghanaian population and further provide evidence of the ameliorating role of fruits and vegetables consumption in the observed relationship. The findings of our study will help better tailor public health intervention strategies for addressing the burden of pregnancy disorders, and PTB and LBW in developing countries. Mothers diagnosed of GDM and HDP should be advised during antenatal care visits to increase intake of fruits and vegetables to help safeguard their health and that of the developing foetus.

### Electronic supplementary material

Below is the link to the electronic supplementary material.

**Supplementary Material 1:** Study Data

## Data Availability

The data that support the findings of this study are included in the article as a supplementary file.

## Abbreviations

APR: Adjusted Prevalence Ratio
CI: Confidence Interval
FFQ: Food Frequency Questionnaire
GDM: Gestational Diabetes Mellitus
HDP: Hypertensive Disorders of Pregnancy
LBW: Low Birth Weight
PTB: Preterm Birth
